# Polarization contrasts and their effect on the gaze stabilization of crustaceans

**DOI:** 10.1242/jeb.229898

**Published:** 2021-04-06

**Authors:** Christian Drerup, Martin J. How

**Affiliations:** 1 CCMAR (Centro de Ciências do Mar), Universidade do Algarve, Campus de Gambelas, 8005-139 Faro, Portugal; 2 Marine Behavioural Ecology Group, Department of Zoology, University of Cambridge, Downing St, Cambridge CB2 3EJ, UK; 3 School of Biological Sciences, University of Bristol, 24 Tyndall Avenue, Bristol BS8 1TQ, UK

**Keywords:** Visual system, Object detection, Motion detection, Contrast vision, Optokinesis, Optomotor reflex

## Abstract

Many animals go to great lengths to stabilize their eyes relative to the visual scene and do so to enhance the localization of moving objects and to functionally partition the visual system relative to the outside world. An important cue that is used to control these stabilization movements is contrast within the visual surround. Previous studies on insects, spiders and fish have shown that gaze stabilization is achromatic (‘colour blind’), meaning that chromatic contrast alone (in the absence of apparent intensity contrasts) does not contribute to gaze stabilization. Following the assumption that polarization vision is analogous in many ways to colour vision, the present study shows that five different crustacean species do not use the polarization of light alone for gaze stabilization, despite being able to use this modality for detecting predator-like objects. This work therefore suggests that the gaze stabilization in many crustaceans cannot be elicited by the polarization of light alone.

## Abstract (precis)

**Summary:** Five different crustacean species do not use the polarization of light alone for gaze stabilization, despite being able to use this modality for detecting predator-like objects.

## INTRODUCTION

Visual systems are crucial for the survival of many organisms and have evolved to produce internal representations of the outside world to facilitate visually guided behavioural tasks. This internal representation is constrained, on the one hand, by the information-gathering capabilities of the visual system, and on the other, by the need to balance physiological, energetic and computational costs. The visual systems of crustaceans can be considered some of the most diverse of all invertebrate groups (Land, 1984). In terms of colour vision, most crustaceans are thought to have monochromatic or dichromatic compound eyes (Cronin and Hariyama, 2002) (with the exception of stomatopods; Marshall et al., 2007). Additionally, a great number of crustaceans possesses the ability to discriminate the polarization of light (Marshall and Cronin, 2014). Briefly, polarization refers to the geometric arrangement of light waves within a beam of light. The degree of polarization (DoP) corresponds to the proportion of light waves oriented in a single plane (varying from 0: unpolarized, to 1: fully polarized), while the angle of polarization (AoP) corresponds to the axis of alignment (ranging between 0 and 180 deg). Light can become polarized after being reflected, refracted or scattered from particles or objects (Cronin and Shashar, 2001; Nilsson and Warrant, 1999; Sabbah et al., 2005). In a marine environment, such objects could be the ocean surface, suspended particles or transparent organisms such as jellyfish or larvae (Cronin and Shashar, 2001). On land, damp or glossy surfaces such as mudflats produce strongly polarized reflections (Zeil and Hofmann, 2001). This property of light is largely invisible to humans (except for Haidinger's brush phenomenon; Haidinger, 1844), but since the discovery of polarization-based orientation in honeybees (Frisch, 1949), this modality of light has been found to underpin a range of behavioural tasks in a wide variety of animal species (Horváth, 2014). Additionally, recent studies on crustaceans have shown that polarization vision tends to be as useful or more so than colour vision in certain habitats (Marshall and Cronin, 2011, 2014).

Crustacean compound eyes are also particularly well tuned for motion vision (Land, 1984). Two well-studied motion vision tasks are gaze stabilization and the detection of moving objects (e.g. prey/predators). To simplify tasks based on motion detection, many animals stabilize their gaze using dedicated eye or body movements which help to avoid motion blur, maintain their orientation with respect to certain landmarks or the horizon, and determine their motion relative to their visual scene (Nalbach, 1990; Zeil and Hemmi, 2006). These particular movements can be considered as optomotor responses (OMR) or optokinetic responses (OKR) (Kretschmer et al., 2017; Land, 1999). While OMR generally refers to head or body movements in unrestrained animals, OKR implies compensatory eye movements in both restrained and unrestrained animals and usually consists of a slow stimulus-tracking phase, followed by a fast saccade-like phase in the opposite direction (Horridge and Sandeman, 1964; Walls, 1962). By exposing an animal to a moving repetitive striped stimulus (grating pattern) and establishing OMR or OKR responses, the limits of this animal's ability to stabilize its visual field can be quantified. Commonly, an ‘optomotor drum’ has been used to determine the acuity limits in various animals, such as mammals (Collewijn, 1969; Prusky et al., 2004), fish (Krauss and Neumeyer, 2003; Roeser and Baier, 2003; Ryan et al., 2016; Schaerer and Neumeyer, 1996), amphibians (Cummings et al., 2008), reptiles (Fite et al., 1979; Fleishman et al., 1997), birds (Fite et al., 1979), insects (Blondeau and Heisenberg, 1982; Borst et al., 2010; Fry et al., 2009; Kaiser and Liske, 1974), spiders (Orlando and Schmid, 2011), cephalopods (Cartron et al., 2013; Darmaillacq and Shashar, 2008; Talbot and Marshall, 2010) and crustaceans (Glantz and Schroeter, 2006; Horridge and Sandeman, 1964; Korte, 1966; Nalbach and Nalbach, 1987).

The detection of moving objects requires motion vision systems to identify moving cues against differently moving or static backgrounds. A common method for investigating object-based motion vision is to expose animals to looming stimuli to elicit a startle response. By varying the parameters of computer-generated, two dimensional stimuli and tracking stereotypical defence and/or avoidance behaviours, thresholds of moving object detection can be determined, as shown in prior work on mammals (Maier et al., 2004; Yilmaz and Meister, 2013), fish (Pignatelli et al., 2011; Preuss et al., 2006), amphibians (Yamamoto et al., 2003), insects (Fotowat et al., 2009; Gabbiani et al., 1999; Rind and Simmons, 1992), cephalopods (Pignatelli et al., 2011; Temple et al., 2012) and crustaceans (Basnak et al., 2018; How et al., 2012, 2014; Oliva et al., 2007; Wilby et al., 2018).

One rarely discussed question in prior studies is whether a species uses the same contrast vision system for eye stabilization and object detection tasks. Experiments on fish (Krauss and Neumeyer, 2003; Schaerer and Neumeyer, 1996), insects (Kaiser and Liske, 1974; Yamaguchi et al., 2008) and spiders (Orlando and Schmid, 2011) have shown that optomotor behaviour in these species is achromatic, or ‘colour blind’, meaning that chromatic contrast alone (in the absence of apparent intensity contrasts) does not contribute to gaze stabilization. As it has previously been assumed that polarization vision is analogous in many ways to colour vision (Basnak et al., 2018; Bernard and Wehner, 1977; How and Marshall, 2014) (with variations in angle and degree of polarization being roughly equivalent to differences in hue and saturation of colour, respectively), this study aimed to test whether gaze stabilization in crustaceans can be driven by polarization contrasts. Here, we show that five different crustacean species cannot use polarization alone for gaze stabilization, despite using this modality for detecting moving predator-like objects.

## MATERIALS AND METHODS

### Ethical statement

The experiments outlined herein were conducted in accordance with UK legislation and with the ethical approval of Animal Welfare and Ethics Review Body at the University of Bristol, under UIN agreement number UB/18/070.

### Animal collection and husbandry

Five crustacean species were used in the present study. Shore crabs, *Carcinus* *maenas* (Linnaeus 1758), were collected from Clevedon Beach, UK (51°26′18.0″N, 2°51′56.7″W). Hermit crabs, *Pagurus bernhardus* (Linnaeus 1758), common prawn, *Palaemon serratus* (Pennant 1777), and rockpool shrimp, *Palaemon elegans* Rathke 1836 were collected from rockpools at Oyster Cove, Paignton, UK (50°25′03.9″N, 3°33′21.6″W). Fiddler crabs, *Afruca tangeri* (Eydoux 1835), were collected from the shores of Río Piedras, El Rompido, Spain (37°13′02.0″N, 7°07′00.0″W) (Table 1). All animals were collected between March and November 2018 and were kept in individual compartments in tanks filled with circulating artificial seawater (Tropic Marin AG, Wartenberg, Germany) or, for *A. tangeri*, in natural seawater from the collection site, at a salinity of 35 ppt and a temperature of approximately 21°C under a natural lighting regime and were fed twice per week.

**Table 1.**
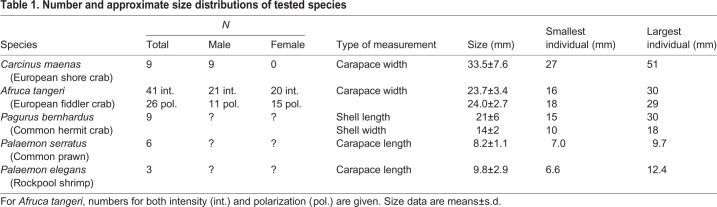
**Number and approximate size distributions of tested species**

### Optomotor/optokinetic arena

**Fig. 1.**
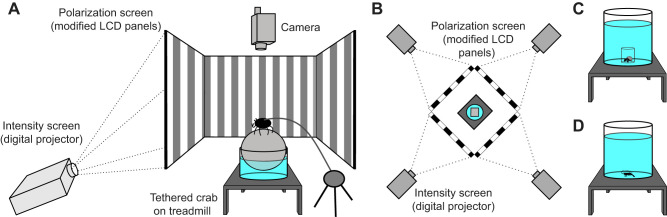
**Diagram of the optomotor arena.** (A) Side view including a tethered *Carcinus maenas* standing on a water-supported treadmill. (B) Top view showing the four digital projectors. (C,D) Schematic diagram of mounting systems for (C) *Pagurus bernhardus* and (D) *Palaemon serratus* and *Palaemon elegans* (referred to in subsequent figures as *Palaemon* spp.).

All species were tested in a virtual optomotor drum, constructed from a combination of modified liquid crystal display (LCD) panels and digital projectors arranged in a four-sided cube to present visual stimuli to the lateral visual field of the subjects. For species obtained from the UK, the optomotor/optokinetic arena was constructed from four custom-built intensity-polarization screens (Fig. 1A,B; for further details about the screen design, see Smithers et al., 2019). Briefly, each screen consisted of two spatiotemporally synchronized displays, (1) a digital projector (CP-WX3541WN, Hitachi Ltd, Tokyo, Japan) that cast an intensity-based image onto (2) a diffuser (250, Lee Filters, Andover, UK) affixed to the rear surface of a 19 inch vertical alignment-type LCD panel dissembled from its outer case (1905FP, Dell Technologies, Round Rock, TX, USA) and with the outermost polarizer removed (Foster et al., 2018). This allowed the simultaneous projection of independently controlled intensity and polarization images to an animal in the centre of the arena (see Fig. S1 for emission spectrum). A simpler field-portable setup consisting solely of four LCD panels (1905FP, Dell) with the outermost polarizer removed was used to test *A. tangeri* near the site of collection in SW Spain. This field apparatus required the addition of external polaroid filters to regain intensity-based images, with a corresponding overall decrease in intensity. The walls of both lab and field arenas had a width of 38.5 cm and height of 30.0 cm, so that they subtended a visual angle of 90 deg horizontally and 82 deg vertically. Intensity contrasts were calculated using the Michelson contrast equation for optomotor gratings and the Weber fraction method for looming stimuli. Full contrast polarization images could be produced with horizontal (H) polarization of DoP=1, and vertical (V) polarization of DoP=0.45. By varying the grayscale value addressed to each pixel (ranging from 0 to 255), different degrees of polarization could be produced (for a full characterization, see Smithers et al., 2019). For all situations where polarization stimuli were presented, a value of polarization distance was calculated based on the method of How and Marshall (2014). Briefly, this method uses the assumption made by Bernard and Wehner (1977) that polarization contrasts are processed in ways that are roughly analogous to colour contrasts. Hence, for a two-channel horizontal versus vertical polarization vision system, we can estimate the amount of activity in an opponent set of photoreceptor cells and use this to model the amount of visual contrast present in polarization between an object and its background (for full methods, see How and Marshall, 2014). In this study, we modelled polarization distance as detected by a standard two-channel polarization vision system with a polarization sensitivity of 10 (for a similar approach, see How et al., 2015; Daly et al., 2016; Smithers et al., 2019). A video camcorder (HC-X900, Panasonic Corporation, Osaka, Japan) was placed above the arena and connected to an external monitor to observe and record the animals without interfering with their behaviour.

Because of the different housing requirements for the five species investigated for this study (Fig. 1A,C,D), different platforms and containers were used to position each animal in the centre of the optomotor arena. *Afruca tangeri* and *C. maenas* were tethered over a treadmill (water- or air-supported polystyrene sphere) by a plastic cable tie fixed to their carapace using a small drop of cyanoacrylate glue (Fig. 1A). This allowed the animals to walk freely while maintaining their body in a stationary position and provided an uninhibited panoramic view of the arena. Crabs inside the arena were wetted periodically with seawater to avoid desiccation. OKR of walking animals (slow stimulus tracking phase of the eyes, followed by a fast saccade-like phase in the opposite direction) was deemed to be the most reliable behavioural response to establish acuity thresholds, as stationary animals occasionally stopped stabilizing their eyes independent of stimulus contrast. For *C. maenas*, this response was determined by eye. For the field data from *A. tangeri*, eye movements were determined using an automated eye-tracking system, custom written in Matlab (2016b, MathWorks, Natick, MA, USA; source code available on request). This system extracted the position of white paint markers on the dorsal carapace cap of each eye to estimate their tracking performance. *Pagurus* *bernhardus* were placed in a small glass cylinder (diameter 3.5 cm, volume 0.05 l) positioned in the centre of a larger glass cylinder (diameter 24 cm, volume 12 l), both filled with seawater (Fig. 1C), allowing them to turn on their body axis within a confined area. Both cylinders were checked for the presence of internal stress regions that may affect the transmission of the polarization of light, but none were found. This species showed barely any OKR in the arena, and because of their small body size and constant antenna and claw movements, eye movement detection became less reliable at lower contrasts. Hence, only OMR (body rotations following the grating pattern) at full contrast were evaluated for the full number of individuals.

Both shrimp species *P. serratus* and *P. elegans* were tested in the above-mentioned water-filled glass cylinder (diameter 24 cm, volume 12 l; Fig. 1D), giving them the possibility to roam freely. As the OKR in both species was difficult to score reliably, only their OMR was evaluated according to the following procedure. When the species followed the grating stimulus continuously by walking around the cylinder for at least one full circuit, the direction of the stimulus was changed, and the response of the animal observed. If the animal followed the grating stimulus again for another full loop, the direction was changed once more, and a positive OMR was noted if the animal again turned to follow the grating pattern.

### Visual stimuli

All stimuli were designed using custom-written software in Matlab (source code available on request). To confirm that each animal was able to respond to both intensity and polarization patterns of the arena, four different full-contrast looming stimuli (black intensity background/white intensity loom and vice versa; horizontally polarized background/vertically polarized loom and vice versa) and one control loom (grey background/grey loom) were presented to each animal and the behavioural responses were observed. The looming stimuli were projected on all four sides of the arena simultaneously and extended within 5 s to a visual angle of 34.5 deg at their maximum following a geometric expansion profile. Each loom was repeated up to 3 times with 1 min intervals and the strongest response per loom was noted in categories from 0 to 3 (see Table 2 for category descriptions). All responses were scored by the same observer.

**Table 2.**
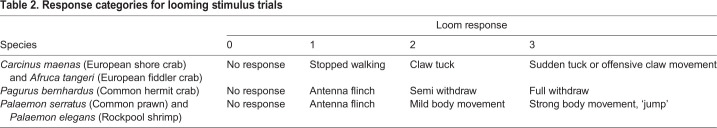
**Response categories for looming stimulus trials**

OMR and OKR responses were determined by casting grating patterns on the four walls of the arena in either intensity or polarization contrast. The grating patterns moved at a fixed rotation speed of 6 deg s^−1^ and the geometry of the pattern was corrected to maintain a constant angular size when viewed from the centre of the arena, thus creating the illusion of a cylindrical optomotor drum. Rotation speed was chosen based on preliminary experiments that showed this to reliably elicit responses to optomotor stimuli. Grating contrast and frequency were systematically varied to determine the OMR/OKR limits of each animal. Frequency values between 0.02 and 0.2 cycles deg^−1^ were picked in a random order. Starting on full contrast, the contrast level was lowered stepwise until no further reaction could be observed. The precise threshold was then determined by narrowing down the contrast level between the last behaviour-eliciting contrast and the first non-behaviour-eliciting contrast. All responses were scored by the same observer.

## RESULTS

### Optomotor/optokinetic responses

**Fig. 2.**
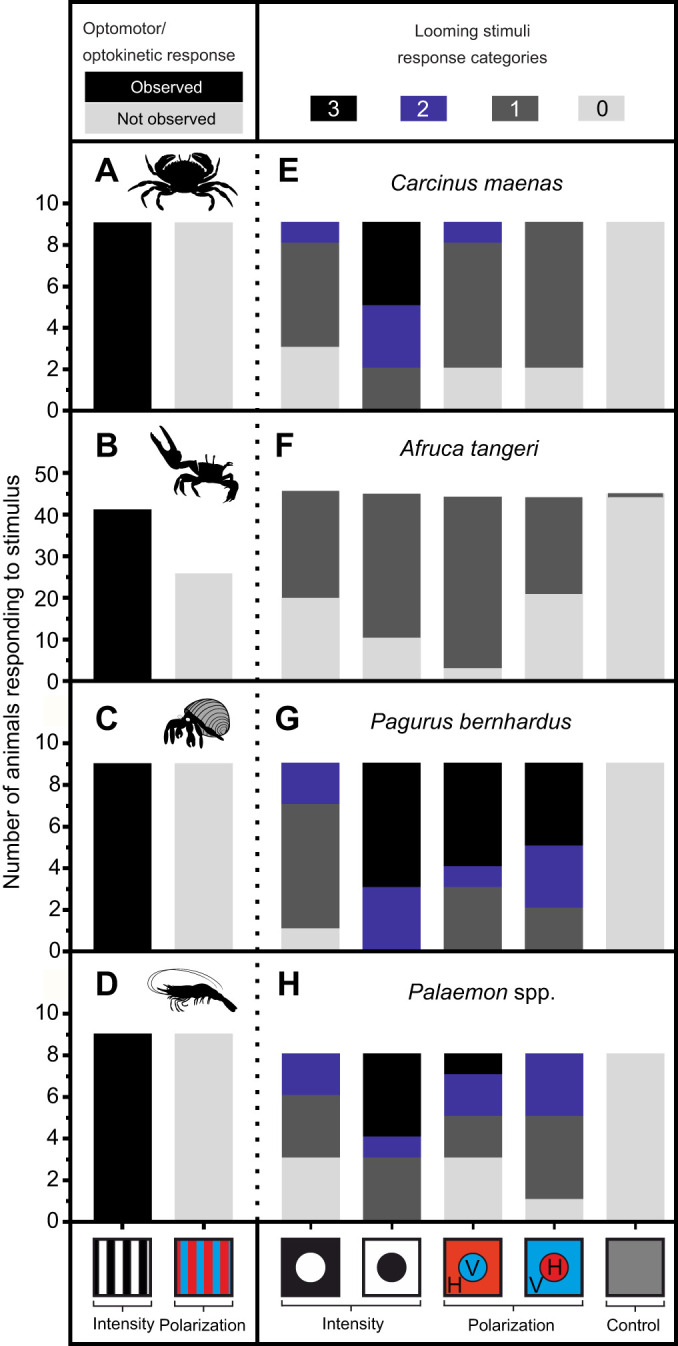
**Optomotor/optokinetic and loom responses of *Carcinus maenas*, *Afruca tangeri*, *Pagurus bernhardus* and *Palaemon* spp.** (A–D) Optomotor/optokinetic responses for intensity and polarized grating patterns. (E–H) Responses to two intensity looms [black background (grayscale value=0)/white loom (grayscale value=255) and vice versa], two polarization looms [horizontally (H) polarized background (DoP=1, AoP=0 deg)/vertically (V) polarized loom (DoP=0.45, AoP=90 deg) and vice versa] as well as one control loom (grey background/grey loom; for each grayscale value=127). Colour is used to represent polarization characteristics, so that red is horizontally polarized and blue is vertically polarized. Response categories go from 3 (strongest response) to 0 (no response). See Table 2 for an exact description of response categories.

**Fig. 3.**
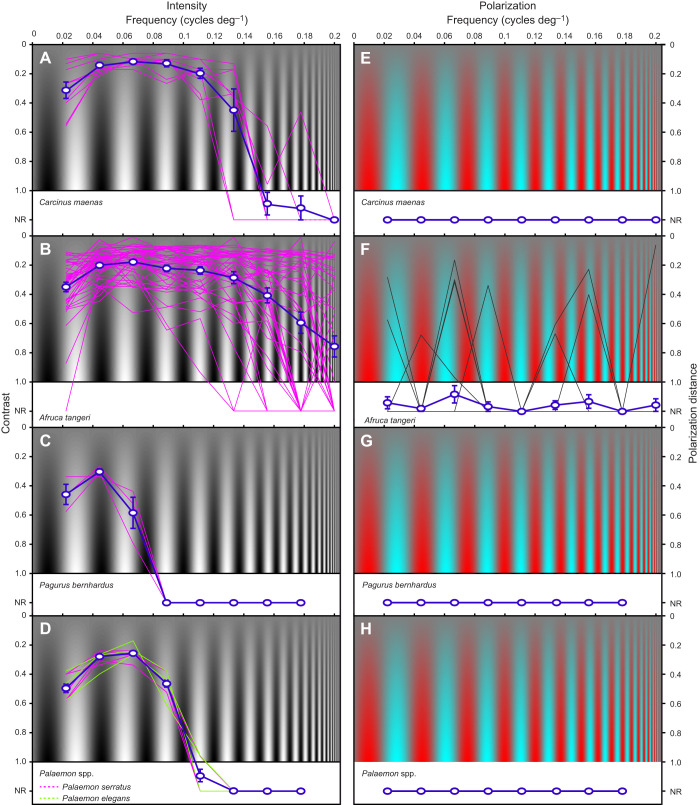
**Contrast sensitivity curves of the tested crustacean species for intensity and polarization.** (A,E) *Carcinus maenas* (*n*=9), (B,F) *Afruca tangeri* (*n*=41 in B, *n*=26 in F), (C,G) *Pagurus bernhardus* (*n*=9) and (D, H) *Palaemon* spp. (*n*=9). Thin lines represent individuals, thick lines represent means±s.e.m. Red background indicates horizontally polarized (DoP=1, AoP=0 deg) and blue background indicates vertically polarized (DoP=0.45, AoP=90 deg). Non-responding animals were assigned an impossible Michelson contrast value of 1.2 (labelled on the *y*-axis as NR for no response) so that the data could contribute to the average response contrast.

Five different crustacean species were tested individually in a virtual optomotor drum. All five species performed OMR or OKR behaviour in response to rotating intensity-based gratings (Figs 2A–D and 3A–D), but none showed any response to gratings based on polarization contrasts alone of any frequency, even when contrast was very high (stripe 1: DoP=1 and AoP=0 deg, versus stripe 2: DoP=0.45 and AoP=90 deg; Figs 2A–D and 3E–H). OMR and OKR to intensity grating patterns varied among tested crustacean species with respect to frequency and Michelson contrast (Fig. 3A–D). As no clear differences between the two closely related shrimp species *P. serratus* and *P. elegans* were found, the two species will be hereinafter referred to as *Palaemon* spp. The frequency response ranged on average from 0.02 cycles deg^−1^, measured in all five species, up to >0.18 cycles deg^−1^ in *A. tangeri* (Fig. 3). The lowest average Michelson contrast values in this study were measured for *C. maenas* (0.11) at a frequency of 0.07 cycles deg^−1^. The OKR of *P. bernhardus* was subtle for weaker stimulus combinations, and so only three complete contrast sensitivity curves could be determined. However, responses to full-contrast stimuli were established across the full frequency range for 9 specimens (Fig. S2). Also note that the data for *A. tangeri* were extracted using an automated eye-tracking system. This system extracted the position of white paint markers on the dorsal carapace cap of each eye. In a small number of cases when eyes were moved in non-tracking behaviours (such as eye cleaning), these produced false positive responses, which caused some level of noise in both intensity and polarization OKR data (Fig. 3B,F). In all cases of polarized optomotor stimuli presented to fiddler crabs, mean response probability was effectively zero.

### Object-based responses

Animals in the same optomotor arena were exposed to a series of looming stimuli to investigate their anti-predator startle responses (Table 2). All five species responded strongly to looms presented in both intensity and polarization contrasts (Fig. 2E–H). Black intensity looms on a white background tended to evoke the strongest response, while the inverse (white intensity loom on a black intensity background) elicited only minor responses. The response of all species towards polarized looming stimuli showed some variation. While most individuals of *C. maenas* and *A. tangeri* exhibited only mild responses to the polarization looms by pausing their movements and only one *C. maenas* exhibited a stronger response (claw tuck-in), several individuals of *P. bernhardus* and *Palaemon* spp. showed stronger responses when exposed to polarization looms, especially to the vertically polarized loom on a horizontally polarized background.

## DISCUSSION

OMR or OKR behaviour was easily elicited in all tested crustacean species when exposed to intensity contrasts, even at average Michelson contrasts as low as 0.11. However, no indication or subtle tendencies of any gaze stabilization movements (locomotor or eye movements) to grating patterns composed solely of contrasts in polarization were observed across the tested species. These findings clearly demonstrate that none of the tested species used the polarization of light on its own to stabilize their visual systems to a rotating visual surround. In contrast, all responded to both intensity and polarized looming stimuli, showing that they were able to use the polarization of light cast by the optomotor arena for a different motion–vision task. We cannot exclude the possibility that polarization contrasts may contribute to enhancing OKR or OMR in more naturalistic settings that contain both intensity and polarization information in combination. This will need to be addressed in future work using mixed intensity/polarization stimuli similar to those used by Smithers et al. (2019).

While there is a multitude of studies demonstrating OMR and OKR to intensity grating patterns in a variety of species, few have attempted to investigate the involvement of polarization contrasts for this behaviour. Glantz and Schroeter (2006) characterized the OKR behaviour of the crayfish *Procambarus clarkii* and *Pacifastacus leniusculus* to gratings projected onto a hemispherical arena. They noted that these species responded well to polarization-only gratings down to contrasts in AoP of approximately 15 deg and DoP of approximately 0.13. Assuming that their experimental setup was well calibrated and free from polarization artefacts, their results suggest that the finding of our current study may not apply to all crustacean species. It must, however, be noted that Glantz and Schroeter’s (2006) stimulus system provided hemispherical cues to the eye of the crayfish, while ours only stimulated the lateral field of view. Perhaps the different observations arise from responses in the dorsal part of the visual field. Indeed, the fiddler crab *A. tangeri* will orient to polarization cues presented to the dorsal eye and perform optomotor behaviour when this pattern is rotated (Altevogt and Hagen, 1964; Korte, 1966). This raises the tantalizing possibility that different visual channels may be employed for similar tasks in different parts of the visual field, a topic that will be of interest for future investigation.

**Fig. 4.**
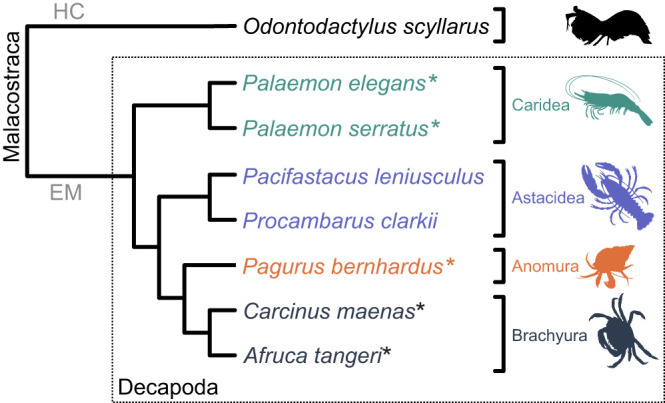
**Crustacean phylogenetic tree.** Schematic illustration of the phylogenetic relationships of the five investigated species (marked with an asterisk) as well as other discussed species in the present study (simplified from Lin et al., 2012; Wolfe et al., 2019). For species of the order Decapoda (dashed box), the corresponding infraorders Caridea, Astacidea, Anomura and Brachyura are shown in different colours. HC, malacostracan subclass Hoplocarida; EM, malacostracan subclass Eumalacostraca.

Following on from this, there could also be subtle differences in the kinds of visual responses that are elicited by rotating optomotor gratings. For example, the crayfish performs a reflex movement towards dorsally presented bright areas, a movement that is also elicited by polarization (Glantz and Schroeter, 2007). Some crustaceans also perform object tracking movements with their eyes (e.g. Cronin et al., 1988; Land et al., 1990; Marshall et al., 2014). While attempting to repeat the current experiment with stomatopod crustaceans (*Odontodactylus scyllarus*; data not shown), we noted that some individuals performed eye movements in response to polarization-only gratings, but we were unable to determine whether these constituted object tracking responses or eye stabilization behaviour. The five species that form the basis of this study do not track objects with eye movements and are more phylogenetically distant from the stomatopods (Fig. 4), which could offer possible explanations for why we saw no response to any of our polarization-based gratings.

Moving beyond crustaceans, there is mixed evidence for polarization-based optomotor behaviour in cephalopods. While no OMR to a pattern of polarized stripes was found in the cuttlefish *Sepia elongata* (Darmaillacq and Shashar, 2008), a positive OMR was found in similar experiments in three closely related cuttlefish species, *Sepia plangon*, *Sepia mestus* (Talbot and Marshall, 2010) and *Sepia officinalis* (Cartron et al., 2013). Darmaillacq and Shashar (2008) assumed that the lack of OMR to the polarization pattern in *S. elongata* might be caused by an insufficient polarization contrast. Interestingly, Cartron et al. (2013) found that polarization sensitivity in *S. officinalis* increases with age. Similarly, orientation responses of fiddler crabs to dorsally presented polarized light could only be elicited in older individuals with a carapace width greater than 7 mm (Altevogt, 1963). In the present study, both juvenile and adult *C. maenas*, *A. tangeri*, *P. serratus* and *P. elegans* were used, whereas only juveniles of the common hermit crab *P. bernhardus* were tested. Hence, the above-mentioned hypothesis could explain the lack of OMR/OKR to a polarization grating for the hermit crab only. However, as all individuals, including those of *P. bernhardus*, responded to at least one polarized looming stimulus, ontogenetic differences with respect to age cannot explain the lack of polarization-based OKR.

While all intensity-based contrast sensitivity curves follow the inverted-U shape observed in a multitude of animals (Harmening, 2017), *C. maenas* and *A. tangeri* outperformed the other three species in both contrast and frequency domains. This difference is most likely down to the larger eyes, which can convey higher levels of resolution and sensitivity (Harmening, 2017; Land, 1984, 1990; Marshall et al., 1999).

The neural substrate for optomotor behaviour has been well characterized in some invertebrate species. In flies, motion vision is dominated by retinula cells R1–6, which feed into two parallel motion pathways in the medulla, one that is sensitive to luminance increments (ON) and one that is sensitive to luminance decrements (OFF) (reviewed by Borst et al., 2020). These motion channels can also incorporate information from receptors R7 and R8, which underpin colour and polarization vision in these species (Wardill et al., 2012). In decapod crustaceans, the seven main photoreceptors R1–7 project to the first two external plexiform layers (epl 1 and 2) where they synapse with three types of descending neuron, two of which carry polarization information, and one that sums the polarization input to convey intensity information only (Strausfeld and Nassel, 1981; Sztarker et al., 2009). The short-wavelength eighth retinula photoreceptor (R8) bypasses the lamina, projecting straight to the medulla. How these various channels of information are then processed for motion vision remains unknown in crustaceans.

**Fig. 5.**
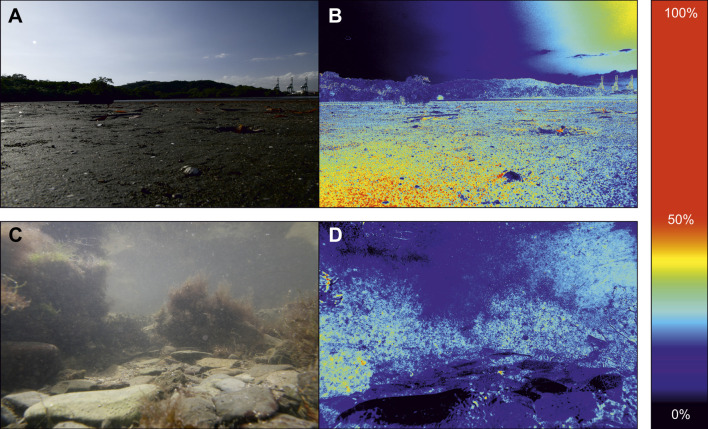
**Photographic polarimetry from an intertidal mudflat and a rockpool.** (A,C) Original colour photograph. (B,D) Corresponding degree of polarization (DoP) image coloured according to the scale (right). See Foster et al. (2018) for methods.

What does this mean in the context of the visual ecology of these species? In some species of crab, OMR and OKR behaviour is largely driven by laterally moving contrasts near the visual horizon (Nalbach and Nalbach, 1987). In uncluttered intertidal environments, such as those inhabited by *A. tangeri*, this region is dominated by the strong intensity contrast between land and sky, an area that shows comparatively little polarization contrast (Fig. 5A,B). Underwater, darker parts of the visual scene appear polarized as a result of scattering from veiling light (Fig. 5C,D) (Schechner et al., 2003), leading to a strong negative correlation between object intensity and polarization. Because intensity alone provides adequate contrast for eye stabilization in both terrestrial and aquatic habitats, perhaps eye stabilization systems would not benefit substantially from the addition of polarization contrast.

In conclusion, the OMR and OKR of five crustaceans were tested to both intensity and polarization grating patterns. While in all species a response was evoked by the intensity pattern, none showed an OMR or OKR to the polarization pattern, suggesting that gaze stabilization in many crustaceans cannot be elicited by polarization alone.
